# A Comparative Clinical and Laboratory Study of Adolescent and Adult Acne in Iraqi Women

**DOI:** 10.7759/cureus.32866

**Published:** 2022-12-23

**Authors:** Samer A Dhaher, Ahmed Hamdan, Zainab T Alyasin

**Affiliations:** 1 Department of Dermatology, College of Medicine, University of Basrah, Basrah, IRQ; 2 Department of Dermatology, Basrah Teaching Hospital, Basrah Health Directorate, Basrah, IRQ; 3 Department of Obstetrics and Gynecology, College of Medicine, University of Basrah, Basrah, IRQ

**Keywords:** hyperandrogenism, hormonal, post adolescent, adolescent, acne

## Abstract

Background

Acne vulgaris is a common chronic inflammatory disease of the pilosebaceous units associated with long-term sequelae and complications. Currently, acne in women is classified into adolescent and post-adolescent forms. However, comparative studies evaluating the clinical and laboratory parameters across various age groups in women with acne are lacking.

The aim of the study is to compare the clinical and laboratory characteristics of different groups of women with acne vulgaris.

Patients and methods

Over 3 years (2018-2021), a cross-sectional study was carried out on 340 women with acne consulting the Dermatology and Venereology Outpatient Clinic, Basrah Teaching Hospital, Basrah, Iraq. Eligible patients were carefully evaluated and fully examined, emphasizing on signs of hyperandrogenism and scoring of acne severity. Hormonal assays of serum total testosterone (TST), dehydroepiandrosterone sulfate (DHEAS), luteinizing hormone (LH), follicular stimulating hormone (FSH), and serum prolactin (PRL) were done. Pelvic ultrasonography was performed to identify any pelvic pathology. The patients were classified according to their age of onset. Clinical and laboratory data were compared among groups.

Results

Three groups were recognized: 160 patients (47%) with adolescent acne (AA) (mean age SD: 17.2±1.6 years), 80 (23.5%) with early adult-onset acne (EA) (mean age SD: 21.4±1.2 years), and 100 (29.4%) with post-adolescent acne (PA) (mean age SD: 28.7±2.9 years), which were further sub-grouped into late-onset acne (40 cases, 11.7%), and persistent acne (60 cases, 17.6%). The mean body mass index was normal in the AA group and overweight in the EA and PA groups. Moderate obesity was more frequent in PA (24%, p=0.03). While 78.5% of AA was mild to moderate acne, 77.5% of EA was moderate to moderately severe, and 72% of PA was moderately severe to severe. Clinical and biochemical markers of hyperandrogenism were seen in all groups, however, they were more frequent in PA and EA groups than in the AA group (p<005).

Conclusion

Clinical and biochemical hyperandrogenism were present in a significant proportion of women with acne; their prevalence was higher in post-adolescent acne than in adolescent acne. Acne that began between the ages of 20 and 25 was classified as "early adult-onset acne," and showed variable features of hyperandrogenism. A complete evaluation, regardless of age, for every female with acne, including a hormonal analysis and pelvic ultrasound examination to detect hormonal imbalances as early therapy, can help to prevent and reduce the risk of consequences.

## Introduction

Acne vulgaris is a chronic inflammatory disease of the pilosebaceous unit, characterized clinically by open and closed comedones, erythematous papules and pustules, and in more severe cases, nodules, deep pustules, pseudocysts, and possible scarring [1]. It primarily affects skin with a relatively high number of sebaceous glands, including the face, upper part of the chest, and back. The resulting appearance can lead to anxiety, reduced self-esteem, and, in extreme cases, depression or thoughts of suicide [2].

The prevalence of acne varies among different age groups, with estimates ranging from 35% to close to 100% of adolescents having acne at some point [3]. In Iraq, acne vulgaris was reported in 293 (7.02%) of 4169 samples, which was graded in the fifth position among all skin diseases [4]. While in Basrah city (southern Iraq), the prevalence in a household survey study was 17% [5]. Four processes have important roles in the formation of acne lesions: alteration of the keratinization of the follicular ostium leading to follicular blockage and comedones formation; increased and altered sebum production by sebocytes under androgen control (or increased androgen receptor sensitivity) leading to hyper-seborrhea and alterations in sebum fatty acid composition; follicular colonization by *Cutibacterium acnes *certain strain with proinflammatory potential; and lastly, the inflammatory mediators released into the skin including IL‑1β, IL‑8, IL‑10 and tumor necrosis factor [6].

Acne can be classified according to severity as mild, moderate, or severe and according to the age of the patient and the onset of the disease [2]. It is common for acne to begin to develop in children between 7 and 11 years of age, often prior to other signs of pubertal maturation. Preadolescent acne tends to be primarily comedonal and favors the forehead and central face ("T-zone") [7]. Polycystic ovary syndrome (PCOS) and other endocrinologic abnormalities should be considered when the acne is unusually severe or accompanied by signs of hyperandrogenism [8]. Post-adolescent acne (adult-onset acne) occurred beyond 25 years of age. It is most common in women and may be associated with a high level of psychological stress [9]. Most affected women present with a mixture of inflammatory and comedonal lesions involving various facial sites and sometimes the trunk, but the mandibular area is involved in 80% of cases. Premenstrual flares are common, but only 20% of women with acne have irregular menses [10].

According to the time of onset, two subtypes of adult women with acne are recognized: persistent acne and late-onset acne. Persistent acne is a continuation or relapse of the disease from adolescence into adulthood and middle age. While the late-onset type involves patients aged 25 years and older who have not previously been affected by acne, late-onset acne is thought to be less common than persistent acne [11]. It is a chronic condition that appears to impact the quality of life in adult patients more than in their younger counterparts, with considerable psychological, social, and emotional impacts and up to a 40 percent prevalence of psychiatric comorbidity [12].

Adult acne pathogenesis involves several endogenous and exogenous factors and may include endocrine disorders, the chronic stimulation of innate immunity, and genetic predispositions; the exogenous factors include cosmetics, stress, and tobacco [13, 14]. Acne-associated hyperandrogenism is suspected in women with irregular menstrual periods, and it is often severe or more difficult to treat, and the onset can be fairly abrupt. Polycystic ovary syndrome (PCOS) is the most common condition associated with elevated serum testosterone, irregular menstrual periods, hirsutism, obesity, insulin resistance, and reduced fertility. To date, the clinical and biochemical characteristics of females with post-adolescent acne have been extensively studied in the current literature; however, comparative studies analyzing the clinical and biochemical features of acne in women of various age groups are lacking.

The aim of the study is to investigate and compare the clinical and biochemical parameters of hyperandrogenism of acne vulgaris in a sample of Iraqi women across various age groups, and to determine whether there was a connection between certain age groups and the severity of the clinical and hormonal profiles.

## Materials and methods

A cross-sectional prospective study was conducted to compare the clinical and laboratory parameters of different groups of women with acne attending the Dermatology and Venereology outpatient clinic at Basrah Teaching Hospital, Basrah, Iraq, over the period from May 2018 to April 2021. The study inclusion criteria were any female patients presenting with acne vulgaris aged 12 years and above. They were divided into different groups according to the age of the onset of acne symptoms. The main exclusion criteria were any patients who had taken any systemic anti-acne medications like anti-androgens, oral contraceptive pills, systemic antibiotics, or isotretinoin for 6 months prior to enrollment. The method of random sampling was employed to collect the patients' data. The sample size was calculated using a simple statistical formula for cross-sectional studies, with a 95% level of confidence and a precision of 5%. We assumed that the prevalence of clinical and biochemical hyperandrogenism was 50% based on the wide range of their prevalence in the available literature (18% to 80%), and a sample size of 176 patients is required for each group.

An anamnesis and careful examination were carried out for each patient. Data was collected using a questionnaire sheet that was arranged after we reviewed the most recent literature. The questionnaire included socio-demographic information, causes and exacerbating factors, first-degree family history, menstrual, gynecological, and obstetrical history, flaring of acne (premenstrual, during menstrual, and post-cycle), and associated symptoms of hyperandrogenism (hirsutism, androgenic alopecia, infertility, and change of voice) and obesity. Hirsutism is defined as excessive growth of terminal hair in the male distribution pattern and scored ≥8 on the Ferriman and Gallwey scale [15]. Androgenic alopecia (female pattern hair loss) is a diffuse central thinning of the crown with preservation of the frontal hairline [16]. Each female was examined for the distribution of acne lesions, whether facial (forehead, cheek, perioral, and lower face) and or extra-facial (trunk, shoulder, neck, and flexural); types of acne lesions, which include non-inflammatory (number of open and closed comedones), and inflammatory (number of papules, pustules, nodules, and cysts); types of scars (pitted, rolling, boxcar, keloid, and hypertrophic). The severity of acne was recorded using the Cunliffe scale as "mild." Comedones (non-inflammatory lesions) are the main lesions. Papules and pustules may be present but are small and few (generally less than 10). Moderate: moderate numbers of papules and pustules (10-40) and comedones (10-40) are present. Moderate-severe: Numerous papules and pustules are present (40-100), usually with many comedones (40-100), and occasionally larger, deeper nodular inflamed lesions (up to 5). Severe: nodulocystic acne and acne conglobata with many large, painful nodular or pustular lesions are present, along with many smaller papules, pustules, and comedones [17]. We calculated BMI and categorized based on WHO 2020 classification [18]. The laboratory workup included hormonal evaluation of serum levels of total testosterone (TST), dehydroepiandrosterone sulfate (DHEA-S), luteinizing hormone (LH), follicular stimulating hormone (FSH), and serum prolactin (PRL) during the follicular phase of the cycle. Ultrasonography of the pelvic organs was performed to detect any pelvic pathology, like polycystic ovary syndrome or ovarian tumors. PCOS was defined according to Rotterdam criteria by the presence of (1) oligo or anovulatory cycles, (2) clinical or biochemical findings of hyperandrogenism, (3) pelvic ultrasound imaging of PCOS [19]. Statistical analysis was performed using SPSS statistics for Windows, version 11.0 (Chicago, USA; SPSS, Inc. software version 11). Results were described as frequency and percentage for qualitative data and mean and standard deviation for quantitative data. One-way ANOVA and Post Hoc Tukey Honest Significant Difference (HSD) (beta) were used to compare the means of three samples. Cross-tabulation was used for comparison between numerical values. A one-sided p-value of <0.05 was considered statistically significant.

## Results

In total, 340 females with various types of acne were included during the study period, and their ages ranged from 14 to 45 years (mean±SD=21.6±5.4 years). According to the age of onset of acne, three groups were identified: adolescent acne (AA, 47%), early adult-onset acne (EA, 23.5%), and post-adolescent acne (PA, 29.4%), which was further divided into two subgroups: late-onset acne (40%) and persistent acne (60%) (Table 1).

**Table 1:** Acne classification of the studied patients according to the age of onset.

Classification	No. (%)	Age of onset (mean±SD)
Adolescent acne (AA)	160 (47)	12-19 years (17.29±1.647 )
Early adult-onset acne (EA)	80 (23.5)	20-25 years (21.45±1.28 )
Post-adolescent acne (PA):	100 (29.4)	>25 years (28.75±2.92)
Late-onset acne	40(40)	
Persistent acne	60(60)	
Total	340	14-45 years (21.62±5.437)

The mean duration of the disease varied depending on the type of acne, as shown in Table 2. A longer duration (55.1±38.7 months) was noticed in PA and was greater than in other groups, with a statistically significant difference (p=0.05).

**Table 2:** Patient demographic of the studied sample.

Variables			Adolescent acne (AA)	Early adult onset acne (EA)	Post-adolescent acne (PA)	P-value
No (%)			160(47%)	80(23%)	100(29.3%)	
Duration (months) Mean±SD			20.34±19.2	36.23±34.65	55.1±38.7	0.05
Family history			104 (65)	52 (65)	62 (62)	0.99
Use of cosmetics			90 (56.25)	64 (80)	80 (80)	0.0098
Body Mass Index (BMI)	Normal		94 (58.75)	34 (42.5)	40 (40%)	0.056
	Overweight		58 (36.25)	34 (42.5)	36(36%)	0.7
	Moderate- obesity		8 (5)	12 (15)	24(24%)	0.03
Mean±SD of BMI			24.1±3.52	25.2±5.59	26.7±3.61	
Fitzpatrick phototype		I	0	2 (2.5)	6 (6)	NA
		II	60 (37.5)	16 (20)	24 (24)	0.058
		III	82 (52.5)	56 (70)	48 (48)	0.4
		IV	16 (10)	6 (7.5)	22 (22)	0.06

A family history of acne in first-degree relatives was found in all groups, and there was no statistical difference between the groups. Patients above the age of 19 in groups EA and PA use cosmetics more frequently than AA patients (80% versus 56.25%), with a highly statistically significant difference (p=0.0098).

The mean level of BMI in all groups was within the normal range, with no statistical difference between groups. However, moderate obesity by BMI was more frequently encountered in PA (24%) compared to other groups, with a statistically significant difference (p=0.03). Regarding the Fitzpatrick phototype of the skin, there was no significant association between the phototypes and any particular group.

The distribution of acne lesions is shown in Table 3. In comparison, the involvement of the forehead and cheek was more often seen in the AA group than in other groups (p=0.05), and the lower face and chin were more frequently involved in the PA group than in other groups (p=0.008), and trunk lesions were more prevalent in the PA group than other groups (p=0.037). Neck involvement was detected only in the PA group (20%).

**Table 3:** The distribution of acne sites and the prevalence of clinical parameters of hyperandrogenism among different acne groups.

Variables		Adolescent (n=160) (AA)	Early adult (n=80) (EA)	Post-adolescent (n=100) (PA)	P-value	
						Facial	Forehead	20 (12.5)	10(12.5)	6 (6)	0.073	
Check	12 (7.5)	16 (20)	12 (12)	0.9		
Forehead and check	104 (65)	26 (32.5)	5 (10)	0.05		
Lower face and chin	24 (15)	28 (35)	72 (72)	0.008		
Extra facial	Trunk	60 (37.5)	22 (27.5)	54 (54)	0.037	
	Shoulder	46 (28.75)	10 (12.5)	14 (14)	0.056	
	Neck	0	0	20 (20)	NA	
	Flexural	0	0	2 (2)	NA	
	Not present	54 (33.75)	48 (60)	10 (10)	0.3	
Seborrhea		138 (86.25)	64 (80)	90 (90)	0.1	
Hirsutism		48 (36.25)	26 (32.5)	50(50)	0.01	
Female pattern hair loss (FPHL)		46 (28.75)	28 (35)	54 (54)	0.005	
Irregular menstrual cycle		50(31.25)	30(37.5)	62(62)	<0.0001	
Premenstrual flaring		48(30)	36(45)	60(60)	0.02	

The prevalence of oily skin (seborrhea) was noticed in all groups, with no statistical difference between them. Hirsutism was found in 36.25%, 32.5%, and 50% of patients in the AA, EA, and PA groups, respectively, and the prevalence in the PA group was higher than in other groups with a statistically significant difference (p=0.01). On the other hand, female pattern hair loss (FPHL) was encountered in all groups but was significantly higher in either the PA or EA groups than in the AA group (p=0.005); however, there was no statistically significant difference between the EA and PA groups.

Although a high percentage of irregular menstrual cycles was found in all groups, it was more frequent in the PA group than in the AA and EA groups (62%, 37.5%, and 31.2%, respectively), with a highly significant association (p=0.0001). Furthermore, the premenstrual flare-up was noticed in a substantial proportion of patients in all groups, and it was significantly higher in the PA group than in other groups (P = 0.02) (Table 3).

Scoring of acne severity showed that the majority of patients with AA (78.5%) had mild to moderate acne, while 77.5% of EA patients had moderate to moderately severe acne, and 72% of PA patients had moderately severe to severe acne, with a statistically significant association between severity score in each group (p=0.0001).

Different types of scars were seen in all groups; overall, 72% of patients in the PA had scars on their faces. while 57.5% of the EA and 31.25% of the AA groups had scars. It is clearly shown that the prevalence of scarring in the PA group was significantly higher than in the EA and AA groups (p=0.02), and in the EA group more than in the AA group (p=0.048). The pitted scar was the most frequent type of scarring in all age groups compared to other scars like rolling or boxcar. There was no hypertrophic scar was noticed in all studied groups. Post-inflammatory hyperpigmentation (PIH) and macular erythema were noticed in all groups, but they were statistically higher in the AA and EA groups than in the PA group (p=0.05) (Table 4).

**Table 4:** The severity of acne and type of scaring among different acne groups.

Variables		Adolescent (n=160)	Early adult (n=80)	post-adolescent (n=100)	P-value
		No. (%)			
Severity	Mild	52 (32.5)	8 (10)	6 (6)	<0.0001
	Moderate	74 (46.25)	36 (45)	26 (26)	
	Moderately-severe	24 (15)	26 (32.5)	32 (32)	
	Severe	10 (6.25)	10 (12.5)	40 (40)	
Types of scar	Pitted	32 (20)	30 (37.5)	36 (36)	0.33
	Rolling	2 (1.25)	4 (5)	16 (16)	0.089
	Boxcar	0	2 (2.5)	2 (2)	0.12
	Mixed	16 (10)	10 (12.5)	18(18)	0.048
	Total No of patients with scar	50(31.25)	46(57.5)	72(72)	0.02
Post-inflammatory hyperpigmentation and macular erythema		42(26.25)	24(30)	22(22)	0.05

The hormonal status of all patients is shown in Table 5. About 52% of the PA, 52.5% of the EA, and 31.25% of the AA groups had an elevation above the normal range of one or more of the measured hormones. There was a significant statistical elevation in the EA and PA groups compared to the AA group (p=0.009). Still, no statistical difference was found between the EA and PA groups (p=0.09). Androgen hormones (TST and DHEA-S) were statistically significantly elevated in the EA and PA groups more than in the AA group (p=0.045); however, there was no statistically significant difference between the EA and PA groups (p=0.07). DHEA-S levels were significantly higher in all groups than TST (12.5% versus 5% in the AA, 40% versus 22.5% in the EA, and 38% versus 28% in the PA group (p=0.0266)).

**Table 5:** Hormonal levels and pelvic ultrasonic examination among different acne groups LH: Luteinizing Hormone, FSH: Follicular Stimulating Hormone, DHEA-S: Dehydroepiandrosterone-Sulphate, PCO: Polycystic ovaries.

Variables		Adolescent Acne No (%)	Early adult Acne No (%)	Post-adolescent Acne No (%)	P-value
Elevated hormones		50 (31.25)	42 (52.5)	52 (52)	0.009
LH: FSH ratio		20(12.5)	20(25)	20 (20)	0.09
S. Prolactin		34 (21.25)	24 (30)	32 (32)	0.07
Total S. testosterone		8 (5)	18(22.5)	28 (28)	0.045
DHEA-S		20 (12.5)	32 (40)	38 (38)	0.026
PCO		52 (32.5)	50(62.5)	50(50)	0.88

A high frequency of PCO was found in all groups. In comparison between all groups, the PCO prevalence was higher with a statistically significant difference in the PA and EA groups than the AA group, but not between the PA and EA groups (p=0.88), as shown in Table 5.

## Discussion

Based on the age at which acne indications first appeared, three patient groups were identified in this study: adolescent acne (AA), early adult onset acne (EA), and post-adolescent acne (PA). After age 25, PA can either develop slowly or late, or it can persist from AA or EA. The majority of patients in this group (60%) had PA that was persistent from AA or EA, which was consistent with the Scroza et al. study, in which 82% of the PA group was persistent acne [11]. 

However, there is still debate over how to categorize women with acne depending on their age of onset. Some authors referred that AA's age of onset may be prolonged to the age of 24 years and PA begun beyond the age of 25 years [19,20], while others viewed the onset of AA as ranging from 12-19 years and PA is typically started beyond the age of 20 years [21]. In the current study, we found that a sizable proportion of patients had acne that started between the ages of 20 and 25 and was categorized into a distinct group known as early adult onset acne (EA). Patients in this group (EA) typically shared the clinical characteristics of AA, including facial features, a low prevalence of clinical hyperandrogenism, particularly hirsutism, FPHL, irregular menstruation, and few scars compared to post-adolescent acne (PA). In contrast, EA acne patients closely resemble post-adolescent acne and have higher rates of biochemical hyperandrogenism and polycystic ovaries than AA. These results might indicate that the location of the acne in this group was intermediate, mimicking both the clinical characteristics of AA and the biochemical parameters of the PA group.

In this study, the mean body weight of the AA group was within the normal range, while that of the EA and PA groups was overweight. Even more so than in other groups, moderate obesity was more prevalent in PA, indicating a possible link between obesity and the emergence of adult acne. Obesity has been shown to increase the secretion of androgens, insulin, insulin-like growth factor 1 (IGF-1), and growth hormones. In acne patients, a correlation between the serum levels of IGF-1, DHEA-S, and dehydroepiandrosterone, as well as the number of acne lesions and sebum production, has also been reported. Acne is indeed a result of IGF-1 activation of 5-reductase, adrenal and gonadal androgen synthesis, and sebocyte stimulation [22].

According to this study, there is a correlation between age and an increase in acne severity and associated problems. Mild to moderate acne was more prevalent in AA, moderate to moderately severe acne was more prevalent in EA, and severe to extremely severe acne was more prevalent in PA. These results support earlier research that has been published [20]. AA is often of intermediate intensity, but PA tends to be more severe and most inflammatory, which may not respond to treatment and raises the risk of scarring. 

We found in this study that clinical symptoms and biochemical indicators of hyperandrogenism were present in all groups, albeit to varying degrees. In the PA group, as opposed to the EA and AA groups, hirsutism, FPHL, irregular menstruation, and premenstrual flare are more frequently observed. There is a statistically significant correlation between hirsutism, FPHL, and the severity and aggravation of acne. This is consistent with Khunger and Kumar's study [20]. Furthermore, the EA and PA groups were found to have higher rates of biochemical hyperandrogenism than the AA group, including higher TST and DHEA-S levels. This finding suggests that the pathogenesis of the EA and PA groups may be more closely related to the hormonal influence of excess androgens than that of the AA group.

LH:FSH ratio and prolactin levels increased across all study groups, although there was no statistically significant difference between them. Khunger and Kumar conducted a hormonal study using serum levels of TST, DHEA-S, LH, FSH, and prolactin (PRL), which was consistent with our findings [20]. The levels of DHEA-S were most often raised in a cross-sectional, retrospective study that evaluated the androgenic hormone profiles of 835 females with acne >15 years of age. Of these, 54.6% had symptoms of hyperandrogenism. A cross-sectional study of Iraqi women with PA revealed that there was an increase in androgen levels in 176 (73.33%) of the patients; of these, 81 patients (34%) only had an increase in DHEA-S, 44 (18%) only had an increase in TST, and 51 (22%) had an increase in both DHEA-S and TST, which is consistent with our study [21].

Regarding PCO, EA and PA groups showed more frequent PCO than the AA group. The same results were mentioned by others, who reported PCO in 52-82% of patients with adult acne [20], and by Borgia et al, who demonstrated PCO in 60 of 129 (46.51%) of patients, but the LH/FSH ratio was increased in only 19 of them, and the ultrasonographic evidence of PCO was not significantly associated with an increased LH/FSH ratio [22]. Gainder and Sharma demonstrated PCO in half of their patients, but the LH:FSH ratio was increased in a third of them [23].

The major limitation of the current study was that our results were based on the finding of a single center (southern Iraq), and the external validity of the study conclusion needs to be confirmed in a larger multi-centric study. Second, although patients on well-known systemic anti-acne medications were excluded from the study, we cannot rule out the potential effect of the long list of topical medications that may have been used by the patients on the signs and symptoms of acne, and this could have confounded the grading of acne severity. Third, the predetermined sample size for each group was not met during the study period, which could be due to a variety of factors, including the emergence of the COVID-19 pandemic and the entire closure at that time, which could confound the study's conclusions. Fourth, other laboratory tests for assessing androgen status, including 17-hydroxyprogesterone, free testosterone, and sex hormone-binding globulin, were not available.

## Conclusions

In conclusion, three main groups of female acne patients were identified: the adolescent acne (AA) group with ages ranging from 12 to 19 years; the early adult-onset acne (EA) group with ages ranging from 20 to 25 years; and the group, which encompasses females above the age of 25 years. Clinical and biochemical evidence of hyperandrogenism was observed in all groups, albeit to varying degrees. Patients with PA tended to be more severe than the other two groups, with considerably greater levels of androgens. EA patients share clinical characteristics with the AA group in that they are mild to moderate in severity as well as have an androgenic profile of post-adolescent acne (PA) with a high prevalence of increased androgens levels. A prospective investigation with a larger sample number of patients is needed to corroborate these findings. Full evaluation, regardless of age, for every female with acne, including a hormonal analysis and pelvic ultrasound examination to detect hormonal imbalances, and early treatment is highly recommended as this will help to prevent the condition from becoming severe and reduce the risk of consequences.
